# Detailing Radio Frequency Heating Induced by Coronary Stents: A 7.0 Tesla Magnetic Resonance Study

**DOI:** 10.1371/journal.pone.0049963

**Published:** 2012-11-21

**Authors:** Davide Santoro, Lukas Winter, Alexander Müller, Julia Vogt, Wolfgang Renz, Celal Özerdem, Andreas Grässl, Valeriy Tkachenko, Jeanette Schulz-Menger, Thoralf Niendorf

**Affiliations:** 1 Berlin Ultrahigh Field Facility, Max-Delbrück Center for Molecular Medicine, Berlin, Germany; 2 Department of Physics, Humboldt University, Berlin, Germany; 3 Siemens Healthcare, Erlangen, Germany; 4 Experimental and Clinical Research Center, a joint cooperation between the Charité Medical Faculty and the Max-Delbrück Center for Molecular Medicine, Berlin, Germany; 5 HELIOS Klinikum Berlin-Buch, Department of Cardiology and Nephrology, Berlin, Germany; University Hospital of Würzburg, Germany

## Abstract

The sensitivity gain of ultrahigh field Magnetic Resonance (UHF-MR) holds the promise to enhance spatial and temporal resolution. Such improvements could be beneficial for cardiovascular MR. However, intracoronary stents used for treatment of coronary artery disease are currently considered to be contra-indications for UHF-MR. The antenna effect induced by a stent together with RF wavelength shortening could increase local radiofrequency (RF) power deposition at 7.0 T and bears the potential to induce local heating, which might cause tissue damage. Realizing these constraints, this work examines RF heating effects of stents using electro-magnetic field (EMF) simulations and phantoms with properties that mimic myocardium. For this purpose, RF power deposition that exceeds the clinical limits was induced by a dedicated birdcage coil. Fiber optic probes and MR thermometry were applied for temperature monitoring using agarose phantoms containing copper tubes or coronary stents. The results demonstrate an agreement between RF heating induced temperature changes derived from EMF simulations versus MR thermometry. The birdcage coil tailored for RF heating was capable of irradiating power exceeding the specific-absorption rate (SAR) limits defined by the IEC guidelines by a factor of three. This setup afforded RF induced temperature changes up to +27 K in a reference phantom. The maximum extra temperature increase, induced by a copper tube or a coronary stent was less than 3 K. The coronary stents examined showed an RF heating behavior similar to a copper tube. Our results suggest that, if IEC guidelines for local/global SAR are followed, the extra RF heating induced in myocardial tissue by stents may not be significant versus the baseline heating induced by the energy deposited by a tailored cardiac transmit RF coil at 7.0 T, and may be smaller if not insignificant than the extra RF heating observed under the circumstances used in this study.

## Introduction

The sensitivity gain and signal-to-noise ratio (SNR) advantages inherent to ultrahigh field (B_0_7.0 Tesla) Magnetic Resonance (MR) hold the promise to enhance spatial and temporal resolution in MR imaging (MRI) [1], [2]. Such improvements could fuel a number of cardiovascular MR (CMR) applications, including the characterization of ischemic and inflammatory disorders on the myocardial tissue level, mapping myocardial microstructure and parametric imaging [3]–[9]. However, intracoronary stents commonly used in percutaneous interventions – a revascularization procedure for treatment of acute and chronic coronary artery disease (CAD) [10]–[12] - are currently considered to be contra-indications for MRI using magnetic field strengths of 7.0 T and higher. This is of profound relevance for the development of CMR applications at ultrahigh fields and their transfer into clinical practice due to an increasing patient population that underwent percutaneous coronary interventions and stent implementation with stent implantation rates of up to 91% [13].

Coronary stents and other implants were carefully examined at lower magnetic field strengths including 1.0 T, 1.5 T and 3.0 T [14]–[21]. At 7.0 T, radiofrequency (RF) heating induced by stents remains a concern due to the relative lack of data. The antenna effect due to the presence of implants with high conductance in conjunction with the decrease in RF wave lengths and the increase in RF energy may cause RF power deposition at ultrahigh fields that may induce local heating [22], [23] and may potentially cause myocardial tissue damage, influence coagulation or affect endothelial function.

Realizing the limits of the International Commission on Non-Ionizing Radiation Protection (ICNIRP) guidelines [24] and its implications for cardiac MRI at 7.0 T this work carefully examines RF induced heating at 7.0 T. To meet this goal a copper tube and coronary stents together with agarose phantoms with electromagnetic properties that mimic myocardium are used. To scrutinize the interference between coronary stents and *E*-fields numerical electromagnetic field (EMF) simulations are performed at a frequency of 297.2 MHz. For this purpose a highpass circular polarized birdcage RF coil is designed to provide RF power well above the limits given by ICNIRP guidelines. To translate the outcome of the numerical simulations into reality, phantom experiments are conducted at 7.0 T using the RF coil design proposed for the numerical simulations together with an RF heating protocol that overrides the clinical local SAR limits by a factor of three. Temperature is monitored throughout the heating protocol using MR thermometry and fiber optic measurements.

**Figure 1:**
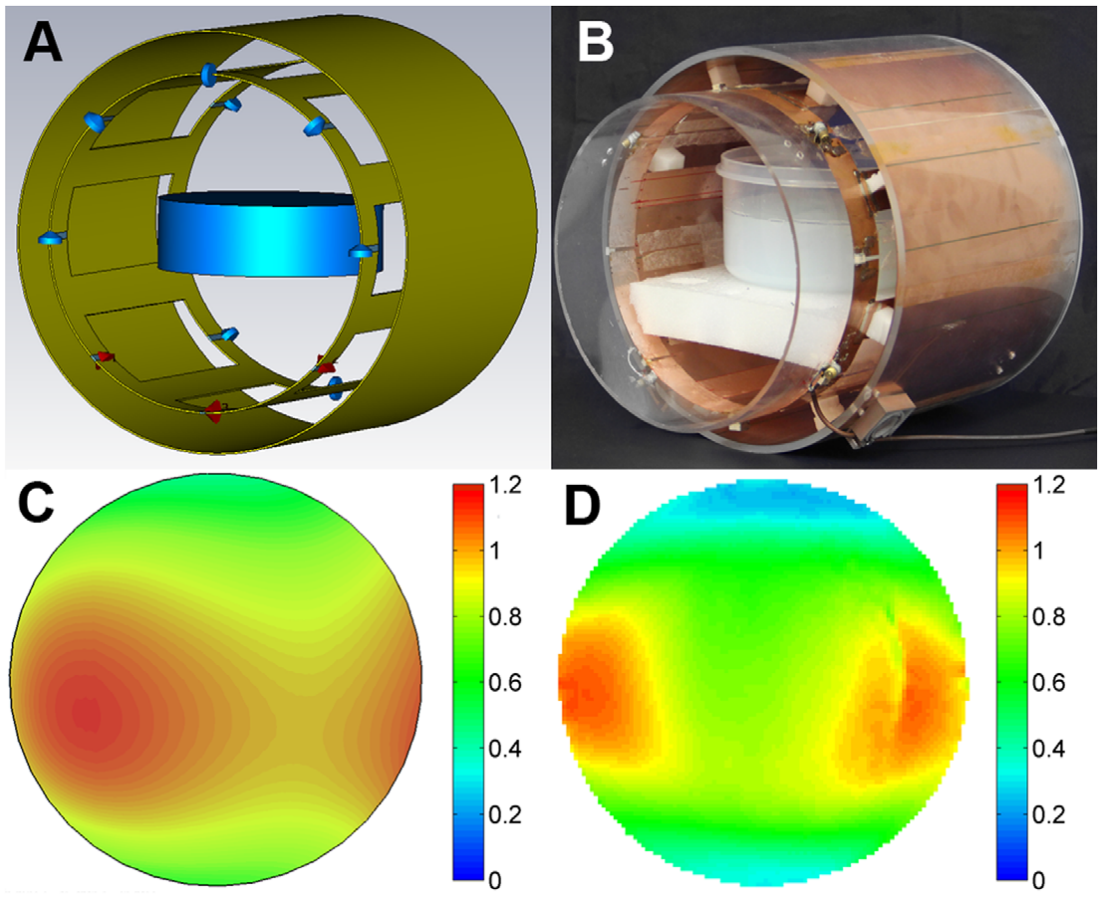
3D model of the birdcage coil used in the EMF simulations (A). The blue surface represents the control phantom positioned in the centre of the coil. The feeding ports (red) and capacitors (blue) located between the rungs are displayed. The middle red port is used for adjusting the transmission coefficients only. **B)** Picture photograph of the experimental birdcage RF coil loaded with the agarose phantom. The inner diameter of the coil and distance between the two end rings is 20 cm. The phantom is fixed with a polystyrene plate in the center of the coil. **C)** Simulated normalized distribution of the *B*_1_^+^ field. Only the partition where the stent has been later positioned is shown. Values are normalized to the experimental nominal *B*_1_^+^. **D)** Experimental normalized *B*_1_^+^ map with nominal *B*_1_^+^ of 20.3 µT corresponding to 22.8 µT/√kW for the slice where the stent has been positioned. *B*_1_^+^ mapping yielded an average *B*_1_^+^ of 11.8 µT/√kW across the target slice, with *B*_1_^+^ being approximately 17.8 µT/√kW at the left side and approximately 11.0 µT/√kW in center of the target slice.

## Materials and Methods

### RF Heating Coil

Radiofrequency power deposited by RF coils approved for clinical MR examinations is limited according to the safety regulations [24] and hence might not be sufficient to induce RF heating. To scrutinize RF power deposition induced heating, an RF heating coil was designed and implemented for EMF simulations and phantom studies. The RF coil exhibits an eight-rung highpass birdcage with feed points at a geometric angle of 0° and 90° with an electrical phase shift of 90°. The inner diameter of the coil is 20 cm. The combined magnetic field of the two ports is a circular polarized *B*_1_ field. To reduce radiation losses and thus increase efficiency, an RF shield was placed around the coil. The basic scheme of the coil design used for EMF simulations is shown in Figure 1A. A picture photograph of the experimental version is depicted in Figure 1B.

**Figure 2:**
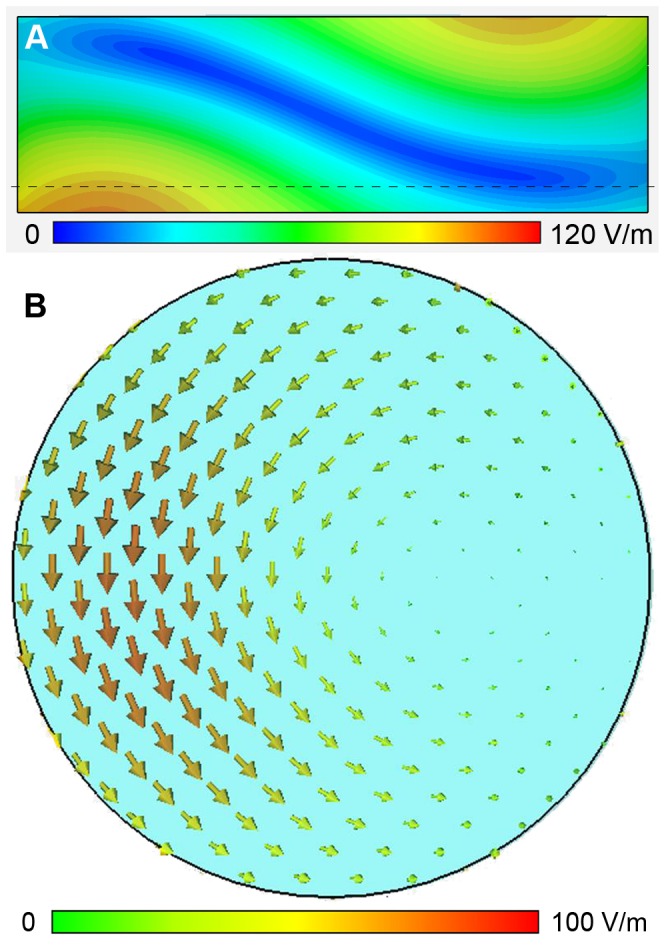
Absolute *E*-field distribution for a transversal slice through the middle of the reference phantom for 1 W rms input power (A). The dotted black line indicates the position of the coronal slice shown in **B.** A maximum *E*-field of 110 V/m is reached at the bottom of the reference phantom in areas closer to the feeding point of the coil. **B)** Vector representation of the *E*-field for a coronal slice marked by the dotted black line in **A.** A phase value of 270° was used in between the feeding points. The vector arrows rotate in the slice plane around its vertical axis of rotation.

**Figure 3:**
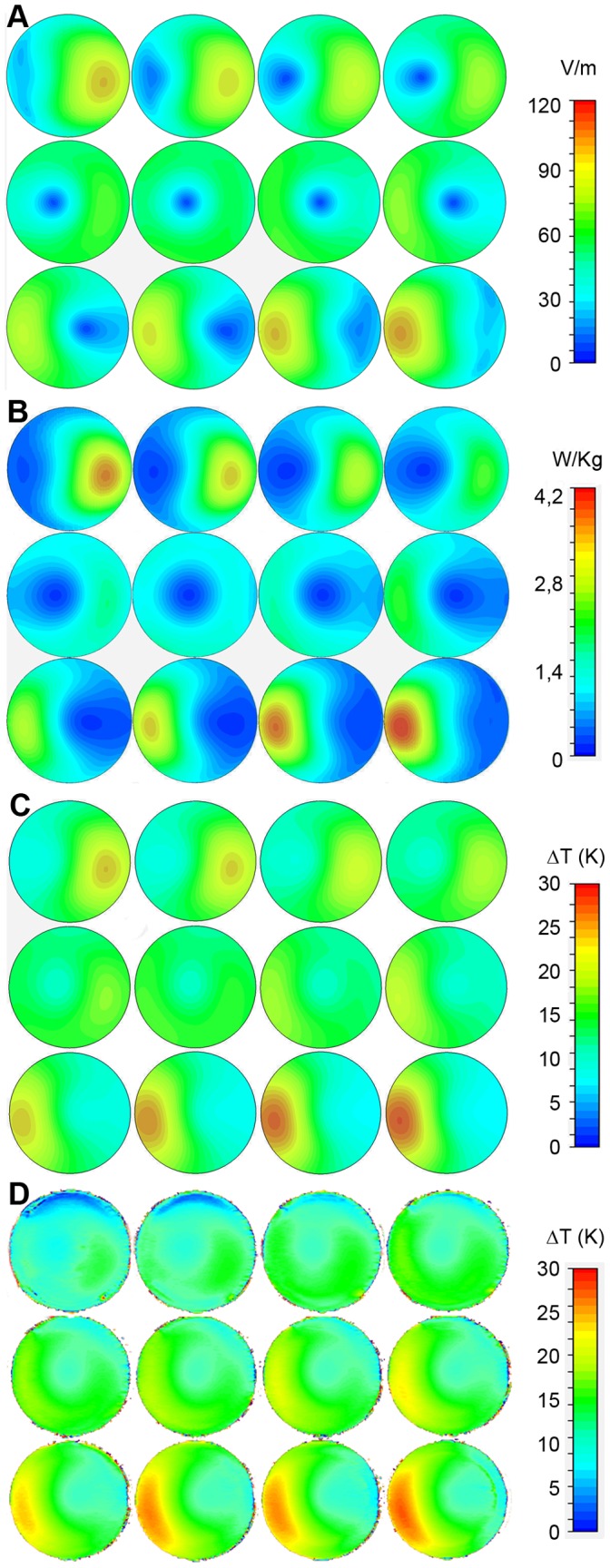
Simulated distribution of the absolute *E*-field in the virtual control phantom for 1 W rms input power (A). The maximum *E*-field was approximately 110 V/m at the bottom slice of the control phantom (slice 12, 3^rd^ row, 4^th^ column). **B)** Simulated distribution of point SAR in the control phantom. SAR follows the *E*-field distribution. Since SAR is proportional to the square of the *E*-field, SAR minima and maxima are pronounced. A maximum SAR of 4.2 W/kg is found for the bottom slice. **C)** Simulated temperature difference distribution in the control phantom. An average RF power of 10 W rms was absorbed in the phantom over 60 minutes. The maximum RF heating induced temperature increase is approximately 29bK in the bottom slice (slice 12, 3^rd^ row, 4^th^ column) and 26 K in the target slice (slice 9, 3^rd^ row, 1^st^ column) of the control phantom. **D)** Experimental temperature difference distribution in the control phantom after one hour of RF heating, obtained with MRth. The smaller heating of the upper slices of the experimental map can be attributed to the lack of insulation on the top of the phantom. The maximum RF heating induced temperature increase is approximately 27 K in the bottom slice (slice 12, 3^rd^ row, 4^th^ column) and 25 K in the target slice (slice 9, 3^rd^ row, 1^st^ column) of the control phantom. A fair agreement between simulated (**C**) and experimental data was found.

### Phantom Design

To measure RF heating, agarose gel phantoms were used which emulate the electrical properties of human myocardium [25] and which mimic the thermal behavior of biological tissue without additional fluid dynamics caused by thermal convection. For this purpose, the phantoms used 4% agarose doped with NaCl (c = 5 g/l) and CuSO4 (c = 1 g/l). A conductivity of = 1.2 S/m was used to match the conductivity of myocardial tissue. This conductivity was determined based on the electrical properties of various body tissues for a broad frequency range [26].

**Figure 4:**
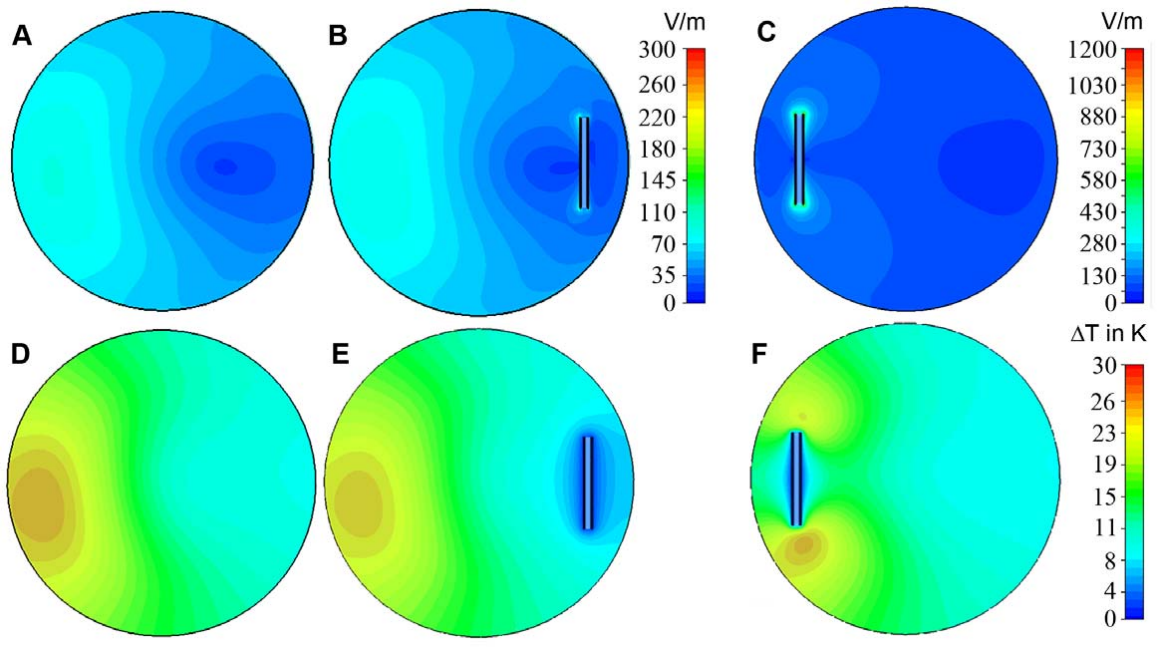
Comparison of simulated *E*-field distributions (A–C) together with simulated temperature difference maps (D–F) obtained for three configurations. A: control phantom only. **B:** phantom with the copper tube positioned in the *E*_min_ region. **C:** phantom with the copper tube placed in the *E*_max_ region. The copper tube couples as an antenna to the electric field which induces *E*-field maxima at the tips and around the center of the copper tube. When the copper tube is placed in the *E*_max_ region (**C**) the *E*-fields at the tip of the copper tube are by a factor of 4 larger versus the configuration where the copper tube has been placed in the *E*_min_ region (**B**). Please note the different scales used in **C** and **B.** The field distortion is also reflected in the temperature distribution. When the copper tube was placed in the *E*_max_ region an extra temperature increase of 3 K versus the heating of the control phantom was found near the tips of the copper tube (**F**). If the copper tube is positioned in the *E*_min_ region (**E**) coupling is weak, and it does not generate any extra heating.

**Table 1:** Synopsis of results derived from numerical EMF simulations.

Configuration	maximum *E*-field (V/m)	maximum temperature increase (K)
control phantom	70 (l), 5 (r)	23 (l), 9 (r)
copper tube in *E*_min_ region	70 (l), 100 (r)	23 (l), 8 (r)
copper tube in *E*_max_ region	1100 (l), 5 (r)	26 (l), 9 (r)

*E*-fields and temperature changes observed for the control phantom and for a copper tube positioned in the lowest *E*-field (*E*_min_) and the highest *E*-field (*E*_max_) regions of the RF heating coil for 1 W rms input power. Values are given for right (r) or left (l) regions close to the position of the tip of the copper tube.

Permittivity was _r_ = 78. To guarantee sufficient loading of the RF coil a cylindrical shape (d = 13.5 cm, volume = 600 ml) was used for all phantoms (Figure 1A, B). An agarose phantom as described is used as a reference and will be referred to as control phantom. Clones of the control phantom were retrofitted with a copper pipe or with a coronary stent. The copper pipe exhibits a length *l* = 40 mm, an outer diameter *d*_out_ = 4 mm and an inner diameter *d*_in_ = 3.8 mm. Two non ferromagnetic coronary stent configurations (PRO-Kinetic Energy Cobalt Chromium Coronary Stent System, Biotronik, Bülach, CH) were used: (i) length *l* = 40 mm, *d* = 4 mm (product # 360553) and (ii) length *l* = 27 mm, diameter *d* = 4 mm (product # 360541).

**Figure 5:**
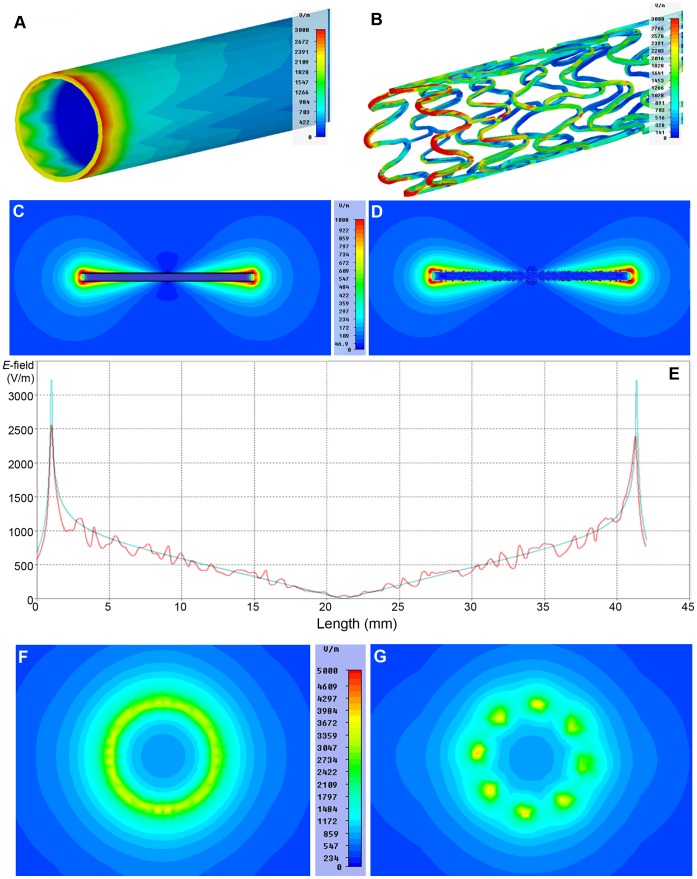
EMF simulations of a copper tube (left) and a stent (right). A uniform *E*-field was generated along the main axis of the devices using an electric dipole excitation. **A, B)** *E*-field at the tips of the devices. **C, D)** Distribution of the *E*-field along the main axis of the devices. The structure of the stent induces small variations of the *E*-field for regions very close to the surface of the stent. **E)** Magnitude of the *E*-field at a distance of 100 m from the surface of the copper tube (cyan line) and the stent (red line). Despite the *E*-field variations induced by the stent design the largest *E*-fields are found near the tips of the stent but are smaller than the *E*-fields at the tip of the copper tube. **F, G)** *E*-fields at the tip of the copper tube (left) and the stent (right) using a view perpendicular to the main axis of the devices. The maximum *E*-field at the tips of the stent is approximately 25% larger than *E*_max_ obtained for the copper tube. The copper tube shows an *E*-field that is more homogeneously distributed along the circular edge.

**Figure 6:**
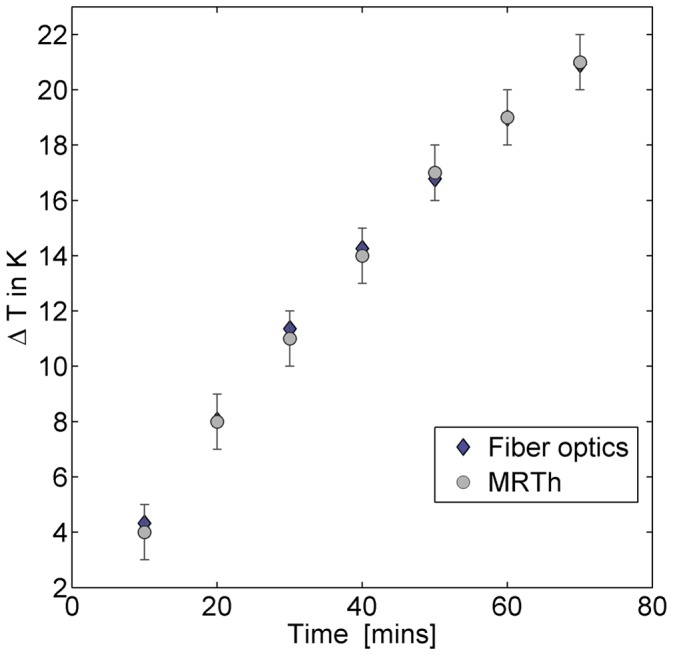
Experimental agreement of the temperature time course measured with MR thermometry versus the fiber optic approach for RF heating in a control phantom over 70 minutes. The experimental error of the fiber optic approach is ΔT = 0.15 K. MRth showed an experimental error of ΔT = 1.0 K. The MRth data were averaged over 25 pixels surrounding the fiber optic probe. For this fiber location a maximum temperature change of ΔT = (211) was observed.

**Figure 7:**
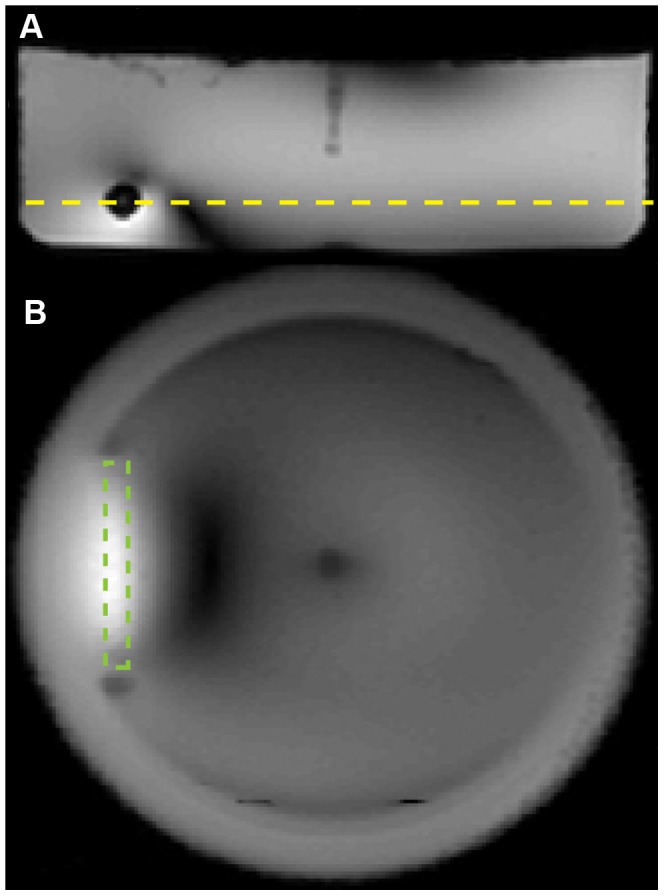
MR localizer showing transversal (A) and coronal (B) views of the agarose phantom together with the position of the copper tube (green dotted line). The yellow dotted line indicates the positioning of the copper tube which corresponds to the positioning used in the numerical simulations (Figs. 2A, 3). Strong MR artifacts are also visible. The same position was used for the stents.

**Figure 8:**
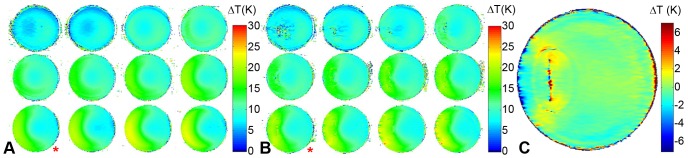
3D delta temperature maps derived from the control phantom (A) and the phantom equipped with the copper tube (B) after one hour of RF heating. In the control phantom the maximum temperature change (ΔT = 27 K) was found in the bottom slice (3^rd^ row, 4^th^ column). The target slice of the control phantom (slice 9, red asterisk) showed a temperature change of ΔT = 20 K in the *E*_max_ region. With the copper tube present in the *E*_max_ region of the target slice a temperature change of ΔT = 23 K was obtained. **C)** Difference of the temperature maps obtained with/without the copper tube for the target slice. Two hotspots are present near the tips of the copper tube with an induced temperature increase of ΔT = (3±2) K.

### Numerical EMF Simulations

Numerical EMF simulations were conducted to characterize the *E*-field distribution of the proposed RF heating coil. This is essential since coupling between objects with high conductance and RF fields strongly depends on the size and position relative to the *E*-field [18], [27], [28]. EMF simulations were also carried out to examine temperature changes induced by the RF heating coil. EMF simulations were performed using the finite integration technique (FIT) (CST software, Darmstadt, Germany) [29], [30]. For this purpose the design, geometry and material parameters of the proposed birdcage RF coil together with the proposed virtual phantom configurations, which match the electrodynamic properties of the experimental phantoms, were applied. A mesh size of (2×2×2) mm^3^ was used for regions inside of the RF coil. For regions inside the phantom around the copper tube a mesh size of (300×700×700) µm^3^ was employed.

**Figure 9:**
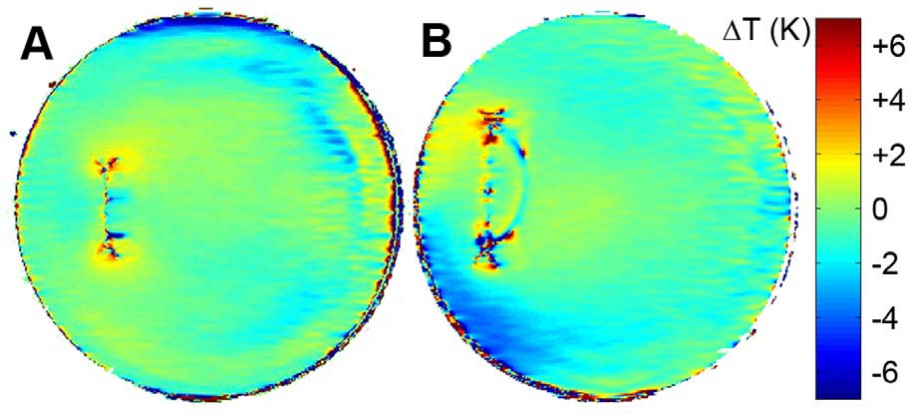
Temperature difference maps between agarose phantoms equipped with coronary stents in the *E*_max_ region (A: *l* = 27 mm, B: *l* = 40 mm) and the control phantom. 60 minutes of RF heating were applied. The maximum temperature difference due to the presence of the stents was found to be ΔT = (32) K for the 27 mm stent and ΔT = (22) K for the 40 mm stent.

**Figure 10:**
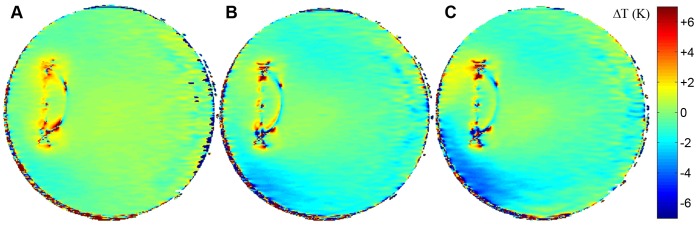
Time course of the temperature difference during an RF heating experiment using an agarose phantom retrofitted with the stent and the control phantom. Delta temperature maps were deduced after 10 minutes (**A**), 40 minutes (**B**) and 60 minutes (**C**) of RF heating. A local temperature increase around the tips of the stents of ΔT = (22) K was observed for all time points.

**Table 2:** Synopsis of the RF heating induced temperature difference near the stent tips after 10, 40 and 60 minutes of RF heating in the phantom equipped with the 40 mm stent.

duration of RF heating (minutes)	control maximum temperature increase (K)	stent local difference intemperature increase (K)	relative local temperature increase control vs stent (%)
10	4±1	2±2	50±50
40	10±1	2±2	20±20
60	24±1	2±2	8±8

The temperature difference between the phantom with the stent and the control phantom (3^rd^ column) remains constant throughout the course of the experiment, even when the absolute temperature increase in the target slice (2^nd^ column) reaches ΔT = 24 K after 60 minutes of RF heating.

To scrutinize subtle details of the *E*-field distribution along the complex structure of the stent high resolution EMF simulations were performed. For this purpose a copper tube and a CAD model of the *l* = 40 mm stent (Biotronik, Bülach, CH) were used together with an electric dipole excitation. The copper tube and the stent were positioned parallel to the electric field vector radiated by the dipole antenna. These simulations were performed with a mesh size of (60×60×60) µm^3^ for regions inside the devices and for regions in the close vicinity of the devices. For the remaining regions inside the phantom a mesh size of (500×500×500) µm^3^ was applied.

EMF and specific absorption rate (SAR) distributions were calculated based on conductivity, permittivity _r_, permeability and density of the phantom and the conducting devices with SAR being proportional to the square of the absolute *E*-field.

Co-simulations using SAR, heat capacity, thermal conductivity of the phantom and a thermal solver of Pennes’ bio-heat transfer equation were conducted. Heat exchange due to blood perfusion was not taken into account in the temperature simulations. Temperature simulations were conducted using an RF power setting which was deduced from preliminary heating experiments. For these experiments an average RF power deposition of about 10 W and accordingly 16 W/kg was assumed inside the control phantom, given a measured RF heating of about ΔT = inside the phantom (mass *m* = 0.6 kg, heat capacity of water *c* = 4184 J/K and duration of 1 h).

### MR Hardware and MR Thermometry

MR experiments were performed on a 7.0 T whole body MR scanner (Magnetom, Siemens, Erlangen, Germany). 3D MR thermometry (MRth) was conducted for temperature mapping [31] using the proton resonance frequency method (PRF) [32]–[34]. PRF produces a map which embodies temperature change Δ*T* relative to an initial temperature. The pulse sequence implemented is a dual echo (*TE*_I_, *TE*_II_) 3D gradient echo (GRE) technique [33]. Change in temperature Δ*T* between time points A and B is derived according to:where α is the temperature-dependent water chemical shift constant in ppm/K, *γ* is the gyromagnetic ratio for ^1^H nuclei, *B*_0_ the static magnetic field, Δ*TE* is the time between the two in-phase echoes. is the difference _BI_-_AI_-(_BII_-_AII_) of the phase images relative to the primary echo (*TE*_I_) at time B minus the same phase image at time A, corrected by the change for the secondary echo (*TE*_II_). This correction factor (_BII_-_AII_) eliminates the dependency of the phase upon changes in electrical conductivity related to temperature [35]. The parameters used for GRE were chosen to accomplish sufficient dephasing between both echoes, without incurring phase wrapping which would be difficult to interpret. A fast repetition time (TR) was used, so that the acquisition time used for temperature mapping is small versus periods of RF heating. The MR thermometry protocol included *TE*_I_ = 2.04 ms, *TE*_II_ = 6.12 ms, *TR* = 10 ms, flip angle = 30 degree, matrix size = 64×64×12 and FOV = (200×140×60) mm^3^. Four oil probes were used as a reference to correct for the *B*_0_ drift [36]. For this purpose the oil probes were placed at a 3 cm distance to the phantom to reduce artifacts near the edges of the phantom due to the oil-water susceptibility difference.

Phantoms were left overnight in the scanner bore to accomplish an equilibrium room temperature prior to the RF heating session. MR thermometry was performed before the RF heating started and every 10 minutes during RF heating. RF heating periods were repeated for one hour.

For validation of MR thermometry, temperature was also measured at four locations of the phantom using a four channel fiber optic system (OpSens, Quebec, CA). This technique is very accurate (0.15 K), offers a sub-second temporal resolution and is immune to interference with magnetic fields. The temperature measurements derived from the fiber optic approach were compared with data obtained from MR thermometry. For this purpose MR temperature maps were averaged for an area of 5 by 5 pixels, all surrounding the fiber optic tip.

A 3D *B*_1_ map of the birdcage coil was acquired at the beginning of the experiment, using the FA CUP method [37], [38] and compared to the simulated one.

### Experimental RF Heating Paradigm

RF heating was achieved by repeating a single pulse experiment. For this purpose SAR monitoring used for human studies was deactivated to allow higher RF duty cycles in conjunction with fast *TR*s. RF heating was performed using: flip angle = 220 degrees, reference voltage V_ref_ = 194 V, pulse voltage V_pul_ = 474 V, τ = 0.5 ms, *TR* = 11.4 ms, number of averages ∼5×10^4^ and TA = 10 min. This setup corresponds to a duty cycle of 4.3% and to a power of 198 W which was estimated under matching conditions and without considering cable, transmit/receive switch and RF coil losses. Such irradiated power is estimated to be about seven times larger than the power used for 2D CINE FLASH based left ventricular function assessment at 7.0 T [39]. For this CMR application a maximum average power of about 30 W [39] was applied while meeting the IEC safety guidelines [40].

However, it is not the irradiated power but the absolute generated *E*-field inside the object which is the primary source of RF induced temperature changes. EMF simulations showed, that the maximum absolute localized *E*-field generated by a TX/RX cardiac coil array at 7.0 T [39], [41] inside the voxel model Duke of the Virtual Family [42] is about ∼430 V/m assuming an input power of 30 W. In comparison, the birdcage coil proposed here generates an *E*-field of ∼985 V/m with an input power of 198 W at the stent position. Consequently this study deliberately uses a local SAR value which is by a factor (985/430)^2^ ≈5 larger than previously reported for an TX/RX array tailored for CMR at 7.0 T [39].

## Results

### RF Heating Coil

Figure 1A shows the basic design of the eight-rung high-pass birdcage coil developed for numerical simulations of RF heating together with the RF-shield and the virtual phantom. The *S*-parameters of the coil were adjusted to maximize absorption at 297.2 MHz. The phase difference between port 1 and port 2 was set to 90 degrees to generate a circularly polarized *B*_1_ field. The experimental version of the RF heating coil is shown in Figure 1B together with the RF shield and the agarose phantom. Matching and tuning parameters were below −35 dB. This setup is able to afford an increased power deposition with a local SAR value (10 g average) of approximately 54 W/kg inside the phantom that exceeds the limits defined by the IEC guidelines [40] by a factor of about three.

A match between the simulated and the measured *B_1_^+^* maps was obtained as illustrated in Figures 1C,1D for a coronal slice of the control phantom which will accommodate the devices. The nominal *B_1_^+^* is 20.3 µT corresponding to 22.8 µT/√kW. *B_1_* ^+^ mapping yielded an average *B_1_* ^+^ of 11.8 µT/√kW across the target slice. At the left side of the phantom *B_1_* ^+^ of 17.8 µT/√kW was found while the center showed *B_1_* ^+^ of 11.0 µT/√kW. EMF simulations, normalized to the same nominal *B_1_* ^+^, revealed a similar *B_1_* ^+^ pattern with a slightly higher *B_1_* ^+^ at the edge and in the central area of the control phantom.

### Numerical EMF Simulations

Figure 2 surveys the *E*-field distribution generated inside the control phantom. The transversal view through the center of the phantom (Fig. 2A) illustrates the *E*-field distribution along the A–P direction, with the *E*-field maxima of 110 V/m at an input power of 1 W rms. To characterize the cross-sectional *E*-field distribution a vector presentation was obtained from a coronal slice as illustrated in Figure 2B. The coronal slice was positioned 10.5 mm away from the coils iso-center. The cross-sectional *E*-field distribution shows an L–R asymmetry. The distribution of the absolute *E*-field across the entire phantom is demonstrated in Figure 3A using a slice increment of 3.6 mm to cover the entire phantom with 12 slices. A maximum *E*-field of 110 V/m was found close to the feeding ports of the coil. The *E*-field shows a conjugate symmetry which reflects the electromagnetic properties of the phantom at 297.2 MHz where the effective wavelength λ_t_ is approximately 12 cm. Please note that slice 9 (3^rd^ row, 1^st^ column) represents the slice used for the *E*-field vector analysis. This slice was chosen to be the target slice for copper tube or stent placement. This approach makes sure that the devices are properly surrounded by agarose. It also helps to reduce the impact of susceptibility gradients at the air/plastic/agarose interface which might hamper MRth.

The target slice yielded an *E*-field L-R asymmetry with the minimum *E*-field of *E*_min_ = 5 V/m being located on the right hand side. A maximum *E*-field of *E*_max_ = 95 V/m was found on the left hand side. This location was used for the stent or copper tube placement. For this purpose the copper tube/stent was strictly aligned parallel to *B*_0_ and to the *E*-field lines using the results obtained from the vector representation of the *E*-field [28] to assure maximum RF coupling between the *E*-field and the copper tube/stent.

Figure 3B demonstrates the point SAR distribution across the entire phantom. For the simulated *E*-fields and SAR distribution the power setting of the feeding ports is normalized to 1 W rms and has to be rescaled for thermal simulations in order to provide comparable data for the experiments. SAR distribution shows a pattern which is similar to the *E*-field distribution, given that SAR is proportional to *E^2^*. Consequently the local SAR maxima/minima are more pronounced versus the *E*-field distribution.

Figure 3C shows the temperature changes (ΔT) induced by the absolute *E*-field and the point SAR distribution. For this purpose a heating period of 60 min was used together with a constant input power corresponding to 10 W absorbed power in the phantom. Please note that the temperature co-simulations consider heat diffusion [43]. This results in a blurring of the areas showing maximum and minimum temperature changes versus the SAR distribution. For the control phantom a maximum temperature change of ΔT_max_ = 29 K was observed, which occurred for slice 12 in Figure 3C. A minimum temperature change of ΔT_min_ = 7 K was found. For the target slice (slice 9) the temperature changes were ΔT_max_ = 26 K and ΔT_min_ = 8 K.

Results derived from EMF simulations involving a copper tube which was placed in the target slice are summarized in Figure 4. Two positions covering *E*_min_ (Figure 4B, 4E) and *E*_max_ (Figure 4C, 4F) were used. When the tube was positioned in the *E*_min_ zone a minor dipole effect was observed for the *E*-fields near the tips of the copper type. For the *E*_max_ region the *E*-field showed a significant antenna effect and reached a maximum of up to 1100 V/m. This *E*-field coupling is reflected in the temperature distributions as demonstrated in Figure 4D–F. Placing the copper tube in the *E*_max_ region induced an additional temperature increase versus the control phantom of approximately ΔT = 3 K near the tips. This corresponds to a relative temperature increase of 13% versus the reference. Positioning the copper tube in the *E*_min_ region caused no significant extra change in temperature versus the control phantom. A synopsis of the results derived from the RF heating simulations is shown in Table 1.

The copper tube provides a reasonable approximation of a coronary stent since its antenna effect is similar to that of a coronary stent as demonstrated by the numerical simulations outlined in Figure 5 for an electric dipole excitation. Figures 5A, B illustrate the *E*-field of the copper tube and of the coronary stent which reaches the maximum at the tips for both configurations. Figures 5C, D show the *E*-field distribution along the main axis of the tube and the stent, which are both aligned along *z* with the external *E*-field. The *E*-field distribution derived for the copper tube and the stent are similar at a scale distance larger than *d*/2. At smaller scales, the structure of the stent induces small variations of the *E*-field which however never exceed the value at the tips, as demonstrated by the *E*-field profiles in Figure 5E. These graphs represent the magnitude of the *E*-field along the main axis of the copper tube/stent at a distance of 100 m from the surface of the devices. Figures 5F, G show the *E*-fields at the tips perpendicular to the main axis of the devices. The maximum *E*-field at the tips of the stent is approximately 25% higher than that of the copper tube, where the *E*-field is more homogeneously distributed along the circular edge.

### RF Heating Experiments

MRth was verified by comparing the temperature time course deduced from MRth with that derived from the fiber optic approach. Figure 6 demonstrates the time course measured in one fiber together with the MRth results averaged over 25 pixels surrounding the fiber. For MRth an error of ±1 K was observed, whereas the fiber optic approach exhibited an error of ±0.15 K.

RF heating experiments using the control phantom revealed a maximum temperature change of ΔT_max_ = (251) K for the target slice as demonstrated in Figure 3D. For the same slice a minimum temperature change of ΔT_min_ = (91) K was derived. For the control phantom a maximum temperature increase of ΔT_max_ = (271) K was found. The relative error of the experimental measurements (Fig. 3D) is below 5% and indicates agreement with the simulation (Fig. 3C). The remaining discrepancy is most likely due to heat diffusion into the air surrounding the phantom. This contribution is not accounted for in the simulation to reduce CPU time.

Figure 7 outlines the position of the copper tube in the phantom which has been positioned 11 mm from the bottom and about 3 cm from the left border of the phantom. The same position has been used for the stents.

The RF heating results are displayed in Figure 8, which shows 3D temperature maps of the control phantom and the phantom containing the copper tube. 3D temperature maps were determined after 60 minutes of RF heating. To highlight the contribution of the copper tube, a temperature difference map of the coronal target slice marked by an asterisk is shown. Similar to the EMF simulations, two hotspots are present near the tips of the copper tube with an extra temperature increase of ΔT = (32) K induced by the antenna effect.

Figure 9 shows the temperature difference maps relative to the control phantom for the two stents. Both revealed hotspots due to the antenna effect, with the hotspot being slightly more pronounced for the 27 mm stent (Fig. 9A). Despite the MR artifact at the position of the stent the temperature difference near the tips versus the control phantom was found to be ΔT = (32) K for the 27 mm stent and ΔT = (22) K for the 40 mm stent.

MRth data were also analyzed for shorter heating periods as illustrated in Figure 10. Despite the large relative error due to the sensitivity of the MRth, the results provide an indication of the coupling behavior of the stent during the course of RF driven heating. The temperature difference maps were obtained after 10, 40 and 60 minutes of heating; a synopsis of the results is given in Table 2. The absolute temperature difference versus the reference phantom is the same for all three time points. This suggests a certain degree of saturation in the heat exchange between the stent and the surrounding agarose phantom. This is indicated by the hotspots being more pronounced after shorter RF heating excitation time.

## Discussion

The results demonstrate an agreement between RF heating induced temperature changes derived from EMF simulations versus temperature maps deduced from MR thermometry at 7.0 T and the measurements with the fiber optic system. The dedicated hardware is capable of irradiating RF power that is by a factor ≈3 above the SAR limits defined by the IEC guidelines. RF heating was performed using a high flip angle FID approach together with short repetition times. Our setup, which comprehends a cylindrical phantom of 600 ml filled with an agarose gel with a conductivity σ = 1.2 S/m and a permittivity ε_r_ = 78 afforded RF induced temperature changes of up to +27 K after a heating paradigm with a duty cycle of 4.3% which was applied with our dedicated RF coil for a period of 60 minutes. The extra temperature increase induced by the copper tube or the coronary stent was found to be about 3 K. For the chromium alloy coronary stent no extra hot spots were evident in the 3D temperature maps in our setup. Please note that blood perfusion will contribute extra cooling for localized heating in *in vivo* experiments.

The coronary stents examined here showed an RF heating behavior similar to that of a copper tube with the same geometry. Unlike the copper tube stents present a network structure in which local hotspots could be present due to parasitic capacitances. Unfortunately MR thermometry is not capable to provide information within the stent network due to the MR artifacts induced by the stent. Here our high spatial resolution EMF simulations provided a better insight into the RF heating pattern inside of a stent.

The maximum temperature changes observed for stents were found to be similar when using a RF heating paradigm for 10 minutes, 40 minutes and for 1 hour. This behavior leads to the hypothesis of a fast saturation of the hotspots and an equilibrium heat dissipation in the agarose. The MRth method implemented here, although prone to some MR artifacts inside the stents and in areas very close to the surface of the stent, has proven valid and reliable in providing 3D temperature maps. As this method relies on the oil references, it is not suitable to measure the temperature change of the oil/fat tissue, and it may be necessary to perfect the method for *in vivo* experiments.

### Conclusions

Our results show agreement between EMF simulations in a virtual phantom and experimental temperature mapping using MR thermometry. The temperature changes induced by RF heating over a one hour period using an RF power, that overrides the regulatory limits, reached approximately ΔT = 27 K in the reference phantom. The extra temperature increase due to the presence of the copper tube or the coronary stents did not exceed 3 K, which is within the experimental error of MRth. It should be noted that, in standard CMR examinations using clinical setups, the actual local RF power deposition will be significantly lower than the one resulting from the RF heating paradigm used here with our phantom setup. Our EMF simulations and phantom studies which exceeded the clinical SAR limits by a factor of three suggest that if IEC guidelines for local and global SAR values are strictly followed, the extra RF heating, induced in myocardial tissue due to the presence of the stent, may not be significant versus than the baseline heating induced by a cardiac optimized transmit RF coil at 7.0 T and may be smaller if not insignificant than the extra RF heating observed under the circumstances used in this study. Before intracoronary stents could be considered safe for CMR and other MRI applications at 7.0 T, *in vivo* validation of the *E*-field estimates and comprehensive examination of clinical CMR protocols are required.
